# Distinct patterns of outcome valuation and amygdala-prefrontal cortex synaptic remodeling in adolescence and adulthood

**DOI:** 10.3389/fnbeh.2015.00115

**Published:** 2015-05-07

**Authors:** Alexandra Stolyarova, Alicia Izquierdo

**Affiliations:** ^1^Department of Psychology, University of California at Los AngelesLos Angeles, CA, USA; ^2^Brain Research Institute, University of California at Los AngelesLos Angeles, CA, USA

**Keywords:** D1 receptors, D2 receptors, PSA-NCAM, stimulus-reward, effort, decision-making, cost-benefit

## Abstract

Adolescent behavior is typified by increased risk-taking, reward- and novelty-seeking, as well as an augmented need for social and environmental stimulation. This behavioral phenotype may result from alterations in outcome valuation or reward learning. In the present set of experiments, we directly compared adult and adolescent animals on tasks measuring both of these processes. Additionally, we examined developmental differences in dopamine D1-like receptor (D1R), dopamine D2-like receptor (D2R), and polysialylated neural cell adhesion molecule (PSA-NCAM) expression in animals that were trained on an effortful reward valuation task, given that these proteins play an important role in the functional development of the amygdala-prefrontocortical (PFC) circuit and mesocorticolimbic dopamine system. We found that adolescent animals were not different from adults in appetitive associative learning, but exhibited distinct pattern of responses to differences in outcome values, which was paralleled by an enhanced motivation to invest effort to obtain larger rewards. There were no differences in D2 receptor expression, but D1 receptor expression was significantly reduced in the striatum of animals that had experiences with reward learning during adolescence compared to animals that went through the same experiences in adulthood. We observed increased levels of PSA-NCAM expression in both PFC and amygdala of late adolescents compared to adults that were previously trained on an effortful reward valuation task. PSA-NCAM levels in PFC were strongly and positively associated with high effort/reward (HER) choices in adolescents, but not in adult animals. Increased levels of PSA-NCAM expression in adolescents may index increased structural plasticity and represent a neural correlate of a reward sensitive endophenotype.

## Introduction

Adolescence is a critical period during which animals learn to predict future states of their habitat depending on current experiences and acquire life strategies that are likely to promote survival and reproductive success later in life. The fitness is increased if the phenotype that developed in early life is matched to the predicted environment (Gluckman et al., 2007), and if an animal can adequately cope with the environmental uncertainty and reward availability (McNamara et al., 2013). Altricial rodents venture out of their home burrow at Post Natal Day (PND) 28, leaving the care of their adult conspecifics, and learn how to acquire nutrients and safety independently (Galef, 1981). The adolescent (PND 28–60) behavioral profile is characterized by increased risk-taking, reward- and novelty-seeking, as well as an augmented need for social and environmental stimulation (Laviola et al., 2003; Kelley et al., 2004; Marco et al., 2011) that may have evolved to promote attainment of the necessary skills for independence (Spear, 2000).

Some of the behavioral patterns common to adolescents across species may result from alterations in reward valuation, marked by an increased sensitivity to reinforcers and reduced sensitivity to costs associated with obtaining them, or stimulus-reward association learning. From a neurodevelopmental perspective, the adolescent period is characterized by pronounced changes in the functional organization and connectivity of the amygdala-prefrontal cortex (PFC) circuit (Cunningham et al., 2002, 2008) and mesocorticolimbic dopamine system (Gelbard et al., 1989; Tarazi and Baldessarini, 2000). Dopaminergic neurotransmission within striatum and PFC is critical to adaptive reward learning and motivation (Berridge and Robinson, 1998; Salamone and Correa, 2002; Cagniard et al., 2006; Ostlund et al., 2011; Salamone et al., 2012; Richard et al., 2013). D1-like (D1R) receptor signaling contributes to cortico-striatal plasticity and regulates reward learning and effort-based decision making (Beninger and Miller, 1998; Baldwin et al., 2002; Schweimer and Hauber, 2006). Similarly, D2-like (D2R) receptor-mediated signaling in striatum has been linked to effort expenditure toward palatable rewards (Trifilieff et al., 2013) and learning from positive outcomes (Groman et al., 2011). The adolescent period is marked by extensive pruning of dopamine D1R and D2R in the striatum (Gelbard et al., 1989; Teicher et al., 1995; Tarazi and Baldessarini, 2000) that may be associated with behavioral differences in reward choices in adolescent vs. adult animals.

Connections between amygdala and PFC are critical for reward responses and choosing between options of different value (Baxter et al., 2000; Blair et al., 2006; Waraczynski, 2006). Structural remodeling within this circuit may be partially dependent on neural cell adhesion molecule (NCAM) function. Previous research has shown that polysialylated NCAM (PSA-NCAM) is critical in synaptic remodeling and plasticity (Muller et al., 1996; Durbec and Cremer, 2001) and modulates cortical neuron sensitivity to neurotrophins (Vutskits et al., 2001). It is expressed in brain regions undergoing structural reorganization during development and in adulthood, including hippocampus, amygdala, and PFC (Nacher et al., 2002a,b; Seki, 2002; Varea et al., 2005). Interestingly, dopamine signaling and PSA-NCAM expression show bidirectional interactions: manipulations of dopamine signaling (systemically and in medial PFC) has been linked to alterations in PSA-NCAM expression (Castillo-Gómez et al., 2008), and a role for PSA-NCAM in dopamine signaling-induced plasticity of PFC inhibitory circuits has also been suggested (Nacher et al., 2013). Similarly, NCAM can promote D2R internalization and subsequent degradation as well as modulate receptor-mediated signaling and behavior (Xiao et al., 2009). PSA-NCAM has already been implicated in learning and stress responses (Pham et al., 2003; Cordero et al., 2005; Bisaz et al., 2009). However, most of the work to date has focused on its role in aversive learning and fear memory, and largely centered on hippocampal function (Senkov et al., 2006; Lopez-Fernandez et al., 2007; Kochlamazashvili et al., 2010). It is not known if PSA-NCAM also contributes to appetitive responses and if the regional specificity of its expression is developmentally specific. This molecular target is of a particular interest as NCAM polysialylation has been linked to neurodevelopmental predisposition to schizophrenia (Hildebrandt et al., 2009), abnormal social interaction and aggression (Calandreau et al., 2010), as well as individual risk for alcohol-related behaviors (Barker et al., 2012). Therefore, in the present set of experiments, we directly compared adult and adolescent animals on tasks measuring both stimulus-reward association learning (Experiment 1) and reward valuation (Experiment 2). Additionally, in Experiment 3, we examined developmental differences in dopamine D1R and D2R expression in striatum and PFC as well as PSA-NCAM expression in PFC and amygdala in adolescent and adult animals trained on an effortful reward valuation task (Stolyarova et al., 2015).

## Material and Methods

### Subjects

Subjects were 24 (Adult = 12, Adolescent = 12) male Long Evans rats (Charles River Laboratories), pair housed. Adolescent animals arrived at our facility at PND 25. They were PND 28 and adult animals were PND 65 at the beginning of handling. Vivaria were maintained under a 12/12 h light/dark cycle at 22°C. All behavioral testing took place 5–7 days a week between 08:00 and 16:00 h during the rats’ inactive period, consistent with previous and ongoing studies in our lab. Research protocols were approved by the Chancellor’s Animal Research Committee at the University of California, Los Angeles.

### Handling and Food Restriction

Rats were left undisturbed for 3 days after arrival to our facility to acclimate to the vivarium. Each rat was then handled for a minimum of 10 min once per day for 5 days. Animals were food-restricted to ensure motivation to work for food for a week prior and during the behavioral testing, while water was available ad libitum. All rats were food restricted based on their baseline food intake that was assessed after the animals had already acclimated to the vivarium to control for the effects of stress on feeding behavior. Food availability was gradually decreased starting with 80% of baseline intake. The amount of food given was never lower than 50% baseline. Weights were monitored daily. We ensured that adult animals did not fall below 85% of their free-feeding body weight and adolescent animals fell within normal age-matched growth weights provided by the vendor. On the two last days of food restriction prior to behavioral training, rats were fed twenty $\frac{1}{2}$ froot loops or sugar pellets in their home cage to accustom them to the food rewards.

### Experiment 1. Reward Learning in Adolescent and Adult Animals

#### Behavioral Apparatus

Behavioral testing was conducted in operant conditioning chambers (Model 80604 Lafayette Instrument Co., Lafayette, IN) that were housed within sound- and light- attenuating cubicles. Each chamber was equipped with a house light, tone generator, video camera, and LCD touchscreen opposing the pellet dispenser. The pellet dispenser delivered 45-mg dustless precision sucrose pellets. Software (ABET II TOUCH) controlled touchscreen stimuli presentation, tone generation, tray- and house-light illumination and pellet dispensation.

#### Behavioral Training

Reward learning was assessed on tasks commonly used as pre-training stages for discrimination learning testing. The training protocol was adapted from Kosheleff et al. (2012) and Izquierdo et al. (2010). Due to a short duration of adolescence in rats (i.e., PND 28–60), only three initial phases were used in the present experiment: Habituation, Initial Touch Training (ITT), and Must Touch Training (MTT). During habituation, rats were required to eat five pellets out of the pellet dispenser inside of the chambers within 15 min before exposure to any stimuli on the touchscreen. ITT began with the display of white graphic stimuli on the black background at the bottom of the touchscreen. The stimuli measured 45 × 45 mm^2^ and were within reach for both adult and adolescent animals. During this stage a trial could be terminated for one of two reasons: if a rat touched the displayed stimulus, or if the stimulus display time (40 s) ended, after which the stimulus was removed and black background displayed. The disappearance of the image was paired with the onset of a “reinforcer event”: dispensation of one (low reward, LR; at the termination of stimulus time) or three (high reward, HR; stimulus touched) sucrose pellets, a 1 stone, and an illumination of the tray-light. Trials were separated by 10 s ITI. In MTT, a trial could be terminated only if the rat touched the image, which then disappeared followed by reward delivery. For both ITT and MTT, the criterion for advancement was set to 60 rewards consumed in 45 min. Animals were given one 45 min session per day until the criterion was reached.

### Experiment 2. Outcome Valuation in Adolescent and Adult Animals

#### Behavioral Testing Apparatus

Rats were tested on a task previously described in Stolyarova et al. (2015) which utilized a maze with three possible courses of action, each associated with different effort requirements and reward magnitudes. Behavioral training and testing were conducted in a standard eight-arm radial maze with arms extending from a central arena with a diameter of 25 cm. Arms were 50 cm long and 12 cm wide. The positions of extramaze cues remained constant throughout all phases of the experiment. The four arms nearest the start arm were permanently blocked, leaving a start arm and three choice arms accessible to animals. One arm of the maze was randomly designated as a low effort/reward (LER), another as a medium effort/reward (MER), and the third as a high effort/reward (HER) arm. The arm assignment was counterbalanced across animals, and held constant between sessions. The barrier heights associated with MER and HER options were adjusted for the present experiment compared to previous study due to reduced ability of adolescent animals to climb over the tallest 30 cm barrier. The arm containing the low reward was unimpeded by a barrier, but in order to obtain the medium or high reward, rats were required to climb a 15 cm or 25 cm barrier, respectively. Rats were required to climb straight up the side (90°) and down at an angle to the food reward located at the end of the goal arm. “Froot loops” (Kellogg NA Co., Battle Creek, MI) were given as food rewards during testing: a “high reward” consisted of four $\frac{1}{2}$ froot loops (i.e., two froot loops), a “medium reward” consisted of two $\frac{1}{2}$ froot loops (1 froot loop), and a “low reward” consisted of one $\frac{1}{2}$ froot loop. Between trials, the rat was removed from the maze and placed in clear Plexiglas holding chamber with a 1651 cm^2^ base and 38.1 cm walls.

#### Habituation

A habituation and training protocol adapted from Walton et al. (2002) was used to habituate the rats to the maze and familiarize them with the froot loops. During the acclimation phase 5 $\frac{1}{2}$ froot loops were placed into each arm of the maze (20 total). Each rat was individually placed into the maze and allowed to explore and eat froot loops freely. Criterion for advancement to the next phase was consumption of 20 $\frac{1}{2}$ froot loops within 15 min.

#### Reward Magnitude Training. Phase 1

In this phase, one goal arm was baited with four $\frac{1}{2}$ froot loops (HR arm), another with two $\frac{1}{2}$ froot loops (MR arm), and the third arm with one $\frac{1}{2}$ froot loop (LR arm). The arm assignment was counterbalanced across animals, and held constant between sessions. Rats were allowed to sample freely from all arms for ten trials. No barriers were present at this phase. Each trial lasted until the rat finished all the froot loops. Trials were separated by a 30 s inter-trial interval (ITI), during which time they were placed in an empty holding chamber. The order of arm visits was recorded. Criterion for advancement to the next phase was completion of ten trials within 30 min.

#### Reward Magnitude Training. Phase 2

This phase was similar to Phase 1 of reward magnitude training, except that animals were allowed to visit only one arm per trial. Rats were removed from the maze as soon as the arm was chosen and the reward was consumed. Animals were given 10 trials per day separated by a 30 s ITI. This phase marked the beginning of learning to visit only one arm as well as continuing to learn each arm’s associated reward values. Criterion for advancement to the next phase was choice of HR arm on 80% or more of the trials for two consecutive days.

#### Alternating Free/Forced Choice Trials with Barriers

During this phase, rats were required to climb barriers to achieve higher rewards. The LER arm continued to be unimpeded by a barrier, but in order to obtain the medium (MER) or high (HER) reward, rats were required to climb a 15 cm or 25 cm barrier, respectively. Thirteen trials were administered per day. Each day of testing consisted of ten free and three forced choice (one for each arm) trials, administered at the beginning. Thus, the structure of the testing was as follows: forced choice trials (1 through 3), followed by ten free choice trials (4 through 13). On forced choice trials all goal arms except one were blocked. The order of arm presentation during forced choice trials was counterbalanced between days. Upon eating the food reward, the rat was placed in a holding chamber for a 30 s ITI, during which the maze was wiped clean with 70% ethanol to prevent the rat’s use of scent-guided choice. Rats were tested daily until stable baseline choice performance was established (choice preferences on free choice trials did not differ across three consecutive days).

### Experiment 3. Amygdala and PFC PSA-NCAM and Striatal D1R and D2R Expression in Adolescent and Adult Animals

#### Tissue Dissection

Rats from ***Experiment 2*** were euthanized 1d after the last day of behavioral testing (late adolescent, PND 50 = 8; adult, PND 86 = 8) with an overdose of sodium pentobarbital (250 mg/kg, i.p.) and decapitated. The brains were immediately extracted and two millimeter-thick coronal sections of frontal cortex, striatum, and amygdala were further rapidly dissected, using a brain matrix, over wet ice at 4°C. Frontocortical dissections included ventral (orbital) and medial sectors of the frontal cortex, but excluded most lateral, posterior (agranular insular) regions. Striatal dissections included both dorsal and ventral subregions.

#### ELISA Method

To prepare the tissues for the assays 0.3 mL (frontal cortex, striatum) or 0.2 mL (amygdala) of PBS (0.01 mol/L, pH 7.2) containing a protease and phosphatase inhibitor cocktail (aprotinin, bestatin, E-64; leupeptin, NaF, sodium orthovanadate, sodium pyrophosphate, β-glycerophosphate; Thermo Scientific, Rockford, IL) was added to each sample. Each tissue was minced, homogenized, sonicated with an ultrasonic cell disrupter, and centrifuged at 5,000 g at 4°C for 10 min. Supernatants were removed and stored at −20°C until ELISA assays were performed. Bradford protein assays were also performed to determine total protein concentrations in each sample. D1R, D2R (Cat# SEB299Ra and SEA673Ra, Cloud-Clone Corp., Houston, TX) and PSA-NCAM (Cat# 67-ABC0027B, ALPCO Diagnostics, Salem, NH) protein levels were determined using a commercially-available ELISA kits. The assays were performed according to the manufacturer’s instructions. The sensitivity of the assays is 0.055 ng/mL for D1R, 0.112 ng/mL for D2R, and 0.25 ng/mL for PSA-NCAM, and the detection range is 0.156–10 ng/mL for D1R, 0.312–20 ng/mL for D2R, and 0.25–16 ng/mL for PSA-NCAM. The concentration of each protein is presented as ng/mg of total protein accounting for dilution factor.

### Data Analyses

Software package SPSS (SAS Institute, Inc., Version 16.0) was used for statistical analyses. Statistical significance was noted when *p* values less than 0.05, and a trend towards significance was noted when *p* values were 0.06–0.08. Days to complete pre-training and establish stable performance were analyzed using *t*-tests. ITT and MIT learning and latency data were analyzed with independent sample *t*-tests and repeated-measures ANOVA (rmANOVA) with trial type as within- and developmental group as between-subject factors where appropriate. The maze choice data were first analyzed using multivariate ANOVA (MANOVA) with barrier height (LER, MER, HER) as within-subject and age group as between-subject factors to probe for differences in choice preferences. When significant interactions were found, *post hoc* simple main effects were reported. ELISA data were analyzed with independent samples *t*-tests.

## Results

### Adolescent Animals Showed Normal Pattern of Growth and Development

**Figure 1:**
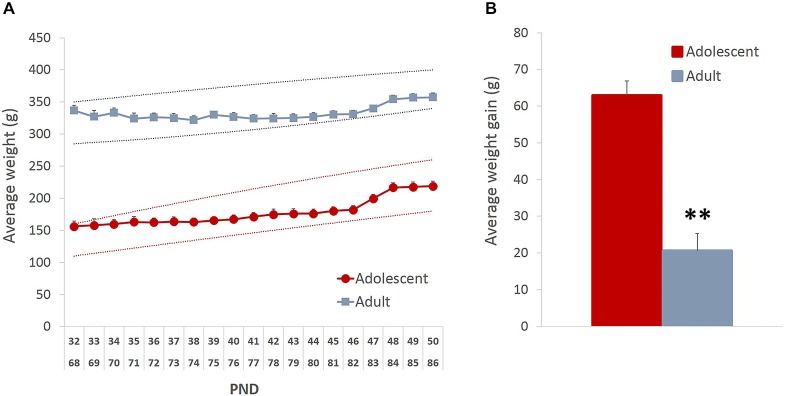
**Mild food restriction did not compromise healthy development of the animals**. The weights were monitored daily from the beginning of food restriction and until the end of behavioral testing.** (A)** Although there was an initial weight loss in adult group (average maximal weight loss = 11% of baseline), both age groups showed an increase in weight by the end of the study. The dashed lines represent the approximate normal growth rate boundaries provided by Charles River Laboratories. **(B)** As expected, the average weight gain in adolescent animals was higher than in adults. Line and bar graphs represent group averages + SEM. **p < 0.01.

To ensure that the mild food restriction did not compromise healthy growth and development of the animals, we analyzed the pattern of weight fluctuations. Although there was an initial weight loss in the adult group (average maximal weight loss = 11% of baseline), both age groups showed an increase in weight by the end of the study (Figure 1A; main effect of time: *F*_(18,396)_ = 30.843, *p* < 0.001), which likely resulted from the supplemental nutrition obtained from the rewards earned during testing. As expected, the average weight gain in adolescent animals was higher than in adults (Figure 1B; *t*_(22)_ = 6.82, *p* < 0.0001).

### Experiment 1. Adolescent Animals are Indistinguishable from Adults in Reward Learning, but Show Distinct Pattern of Responses to Differences in Outcome Values

**Figure 2:**
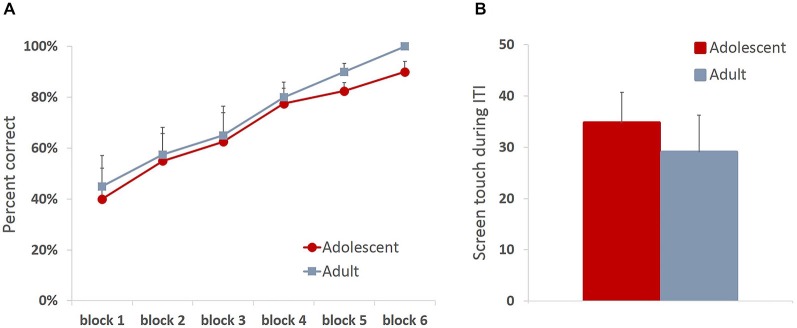
**Adolescent animals are indistinguishable from adults in stimulus-reward appetitive learning**. All animals readily completed initial touch training (ITT) and must touch training (MTT). Adolescent and adult animals mastered the task at a comparable rate, there were no group differences in sessions to criterion on either of the tasks** (A)** All animals improved their performance within session with no differences between age groups **(B)** Adolescent and adult animals learned the contingency of reward on the stimuli, demonstrating low levels of nose-poking the screen during the ITI with no group differences. Line and bar graphs represent group averages + SEM.

**Figure 3:**
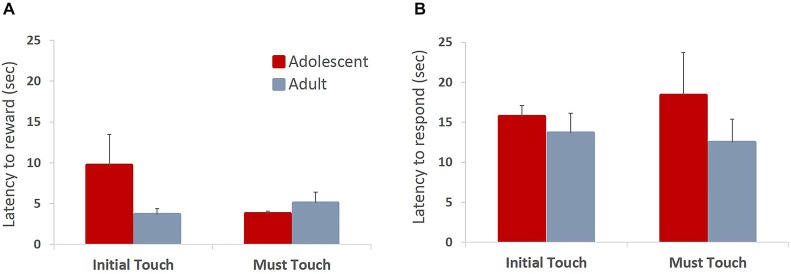
**Latencies to collect reward and respond on the subsequent trial in operant stimulus-reward association learning tasks**. Bar graphs represent mean latency (sec) to collect reward following a correct response **(A)** or touch the stimulus on the subsequent trial **(B)** + SEM. There were no differences in latencies to collect reward or touch the stimulus between the two age groups in either of the training stages. Although adolescent animals were faster to collect reward during the later stage of the training, this difference did not reach statistical significance.

All animals readily completed ITT and MTT. ITT criterion was reached in one session by the majority of the animals, whereas the range for MTT completion was 1–3 sessions. Adolescent and adult animals mastered the task at a comparable rate, there were no group differences in sessions to criterion on either of the tasks (MTT: *t*_(6)_ = 0.361, *p* = 0.73), or percent correct in ITT task (*t*_(6)_ = 0.678, *p* = 0.523), suggesting that adolescent animals are not different from adults in acquiring stimulus-reward contingencies. To verify that the apparent lack of differences in learning rates between age groups were not due to ceiling effects, we analyzed learning progression within session by comparing performance in blocks of 10 trials. All animals improved their performance within session (Figure 2A; main effect of trial block: *F*_(5,30)_ = 24.781, *p* < 0.0001) with no differences between age groups: no main effect of age (*F*_(1,6)_ = 0.249, *p* = 0.635) or age × trial block interaction (*F*_(5,30)_ = 0.159, *p* = 0.75). To establish that animals learned the contingency of reward on the stimuli, we also analyzed animals’ responses during the ITI intervals. All animals showed low levels of nose-poking the screen during the ITI, and there were no differences between adult and adolescent animals (Figure 2B; *t*_(6)_ = 0.614, *p* = 0.562). There were no age group differences in number of food magazine entries in ITT (mean adolescent = 351; mean adult = 329.5; *t*_(6)_ = 0.375, *p* = 0.721). However, adolescent animals demonstrated increased reward-seeking behavior (mean number magazine entries = 188.54) in MTT compared to adults (mean = 138.5; *t*_(6)_ = 3.128, *p* < 0.05). Latencies to collect reward and touch the next stimulus after reward receipt have been suggested to index sensitivity to reinforcing properties of the reward. Overall, there were no differences in latency to collect reward (Figure 3A; ITT: *t*_(6)_ = 1.576, *p* = 0.166; MTT: *t*_(6)_ = 0.919, *p* = 0.394) or touch the image (Figure 3B; ITT: *t*_(6)_ = 0.726, *p* = 0.495; MTT: *t*_(6)_ = 0.958; *p* = 0.375) between the two age groups. Although adolescent animals were faster to collect reward during the later stage of the training (Figure 3A) this difference did not reach statistical significance: no main effect of time (*F*_(1,6)_ = 1.274, *p* = 0.302) or time × group (*F*_(1,6)_ = 3.36, *p* = 0.117) interaction were observed.

**Figure 4:**
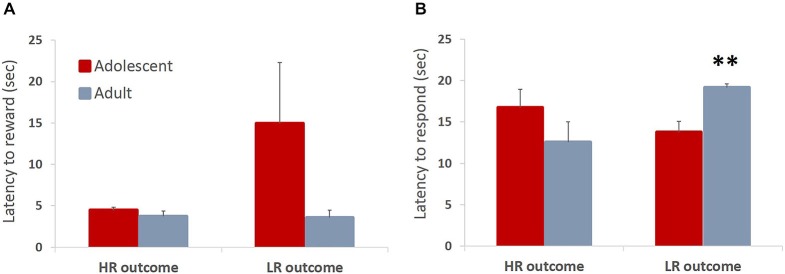
**Latencies to collect reward and respond on the subsequent trial depending on the outcome of the previous trial**. Bar graphs represent mean latency (sec) + SEM. In the ITT phase of learning, animals can receive a reward of larger magnitude (HR; three sugar pellets) upon touching the stimulus, or a low reward (LR; one sugar pellet) non-contingently if they fail to touch the stimulus within allotted time. The magnitude of the received reward could affect subsequent behavior. **(A)** Adolescent animals were slower to collect the HR that was contingent on their response compared to the “free” LR that was delivered at the end of the trial if the animals failed to respond, although this difference did not reach statistical significance. **(B)** Adolescent animals were significantly faster to respond on the subsequent trial after receiving the LR compared to adult animals. Whereas there was a tendency for invigoration of response following lesser-than-expected outcome in adolescent animals, adult animals were slowed by such outcomes. **p < 0.01.

In the ITT phase of learning, animals can receive a reward of larger magnitude (HR; three sugar pellets) upon touching the stimulus, or a low reward (LR; one sugar pellet) non-contingently if they fail to touch the stimulus within allotted time. Therefore, the magnitude of the received reward could affect subsequent behavior. We hypothesized that animals would be faster to collect larger rewards respond on subsequent trials after receiving the large reward. Contrary to our hypothesis, there were no differences in latencies to collect rewards between adolescents and adults. Adolescent animals were somewhat slower to collect the HR that were contingent on their response compared to the “free” LR that was delivered at the end of the trial if the animals failed to respond, although this difference did not reach statistical significance: no main effect of trial type (*F*_(1,6)_ = 2.076, *p* = 0.2) or trial type × age group interaction (*F*_(1,6)_ = 2.178, *p* = 0.19) were observed (Figure 4A). Thus, the outcome of the previous trial did not have an effect on the latency to collect the reward (all *p* values > 0.05). We did, however, observe a between-group difference in the latency to touch the stimulus depending on the outcome of the preceding trial: trial type × age group interaction (*F*_(1,6)_ = 8.105, *p* = 0.029). Adolescent animals were faster to respond on the subsequent trial after receiving the LR compared to adult animals (Figure 4B; *p* < 0.05). This may suggest that adolescent animals were more sensitive to receiving reward of a lesser magnitude than expected (negative contrast). It needs to be noted that in the ITT task animals responded to the stimulus in the majority of trials (mean adolescent 68%, mean adult 74%), therefore delivery of HR was a rule, rather than an exception. The pattern of observed responses suggests that animals respond differently to negative contrasts depending on their developmental stage. Whereas there is a tendency for invigoration of response following lesser-than-expected outcome in adolescent animals, possibly in an attempt to increase overall number of rewards collected, adult animals are slowed by such outcomes.

In summary, the present results revealed a lack of developmental differences in stimulus-reward association learning: adolescent animals were as fast as adults to master the task and direct their responses toward the relevant stimuli. However, these results suggest developmentally different responses to differences in outcome values, which may manifest in a distinct pattern of choices in tasks measuring motivation to obtain rewards (***Experiment 2***).

### Experiment 2. Adolescent Behavior is Characterized by Increased Motivation to Obtain Larger Rewards Over Less Valuable Freely Available Options

**Figure 5:**
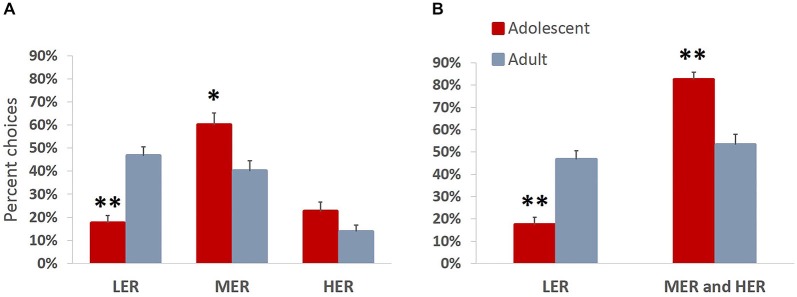
**Increased motivation to obtain larger rewards over less valuable freely available options in adolescent animals**. Bar graphs represent percent high effort/reward (HER), medium effort/reward (MER), and low effort/reward (LER) choices + SEM. Animals were trained on a task allowing them to choose between three available courses of action, each associated with different effort requirement and outcome values. **(A)** Adolescent animals chose significantly less LER and more MER compared to the adult group. Adult animals demonstrated reward devaluation as a function of increases in barrier heights. This trend was absent in the adolescent group, which showed a clear preference for MER over LER and HER options. **(B)** MER and HER arm choices were combined to evaluate preference for easily available over effortful options associated with greater reward magnitudes. Adolescent animals chose significantly less LER and significantly more MER and HER compared to adults. Notably, the preference for effortful options associated with greater reward magnitudes over less profitable freely available options was present in the adolescent but not the adult group. *p < 0.05, **p < 0.01.

We were interested in directly comparing developmental differences in animals’ motivation to obtain rewards of different magnitudes. Animals were trained on a task allowing them to choose between three available courses of action, each associated with different effort requirement and outcome values. This task is more ecologically valid and has been previously shown to effectively reveal differences in reward valuation following methamphetamine treatment, that are not easily observed in a typical T-maze effort discounting task (Stolyarova et al., 2015). In accordance with ***Experiment 1***, there were no group differences in the number of sessions that the animals required to complete pre-training (mean adolescent = 5.63; mean adult = 5.75) or reach stable performance (mean adolescent = 5.13; mean adult = 4.75; all *p* values > 0.39). An omnibus ANOVA revealed a significant main effect of age group on choice preferences across reward arms (*F*_(3,12)_ = 10.963, *p* = 0.001, Wilk’s Λ = 0.267). A significant effect of age group was observed for LER (*F*_(1,14)_ = 34.717, *p* < 0.001) and MER (*F*_(1,14)_ = 8.262, *p* = 0.012), but not HER (*F*_(1,14)_ = 3.053, *p* = 0.102) choices, with adolescent animals choosing significantly less LER and more MER (Figure 5A) compared to adult group. Adult animals distributed their choices uniformly between LER and MER goal arm options (*p* = 0.421), but chose significantly more LER (*p* < 0.001) and MER (*p* = 0.005) compared to HER, demonstrating reward devaluation as a function of increases in barrier heights. This trend was absent in the adolescent group, which showed a clear preference for MER over LER (*p* = 0.001) and HER (*p* = 0.004) options. When MER and HER arm choices were combined to evaluate preference for easily available over effortful options associated with greater reward magnitudes, an age group difference was observed: adolescent animals chose significantly less LER and significantly more MER and HER compared to adults (*p* < 0.001). Notably, the preference for effortful options associated with greater reward magnitudes was present in adolescents (*p* < 0.001) but not the adult group (*p* = 0.407) (Figure 5B).

**Figure 6:**
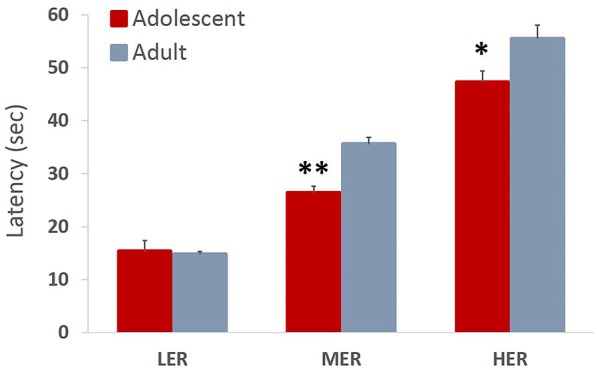
**Trial latencies in an effortful reward valuation task**. Bar graphs represent average latency (sec) to complete the trial + SEM. Trial latencies increased with barrier height in both adolescent and adult animals. Adolescent animals were faster to complete MER and HER trials compared to adult group. *p < 0.05, **p < 0.01.

Trial latencies increased with barrier height in both adolescent and adult animals (all *p* values < 0.01). Omnibus analysis revealed a significant main effect of age group on trial latencies across reward arms (*F*_(3,12)_ = 6.944, *p* = 0.007, Wilk’s Λ = 0.346). A significant effect of age group was observed for MER (*F*_(1,13)_ = 22.841, *p* < 0.001) and HER (*F*_(1,13)_ = 6.022, *p* = 0.029), but not LER (*F*_(1,13)_ = 0.224, *p* = 0.643) choices, with adolescent animals taking significantly less time to complete MER and HER trials compared to adult group (Figure 6).

### Experiment 3

#### Adolescent Animals Show Lower Levels of D1, but not D2 Receptor Expression in Striatum

**Figure 7:**
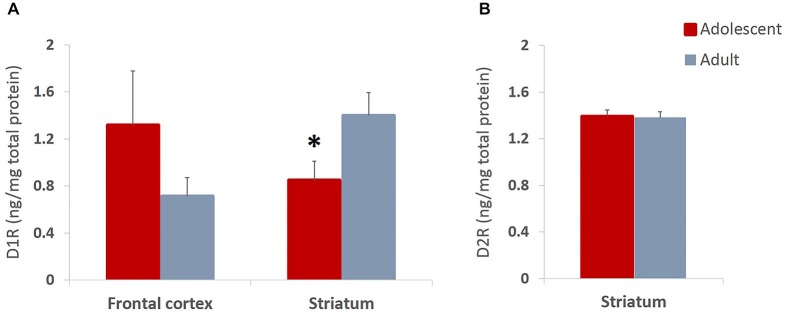
**Dopamine D1 and D2 receptor expression in adolescent and adult animals**. The bar graphs represent the concentration of D1R **(A)** and D2R **(B)** protein (ng/mg of total protein) + SEM. **(A)** D1R expression was non-significantly increased in frontal cortex and significantly reduced in striatum of animals that had experiences with reward learning during adolescence compared to animals that went through the same learning in adulthood. **(B)** The levels of D2 receptor expression were not different between the animals that underwent behavioral testing during adolescence or adulthood. *p < 0.05.

We found non-significant increases in expression of D1 receptors in frontal cortex and significantly reduced D1 receptor expression in striatum (*t*_(14)_ = 2.204, *p* = 0.045) of late adolescent compared to adult animals (Figure 7A). Importantly, this is consistent with previous reports which indicate that animals in early adolescence show greater levels of D1 receptors that are gradually pruned away by early adulthood (Teicher et al., 1995; Tarazi and Baldessarini, 2000). Therefore, the present results suggest that the reward experiences in adolescents may exaggerate normal pruning patterns and result in lower D1R levels as compared to the same experiences encountered in adulthood. The levels of D2 receptor expression in the striatum were not different between the animals that underwent behavioral testing during adolescence or adulthood (Figure 7B).

#### Increased Expression of PSA-NCAM in Adolescent Animals is Associated with Greater Effort Expenditure Toward Larger Rewards

**Figure 8:**
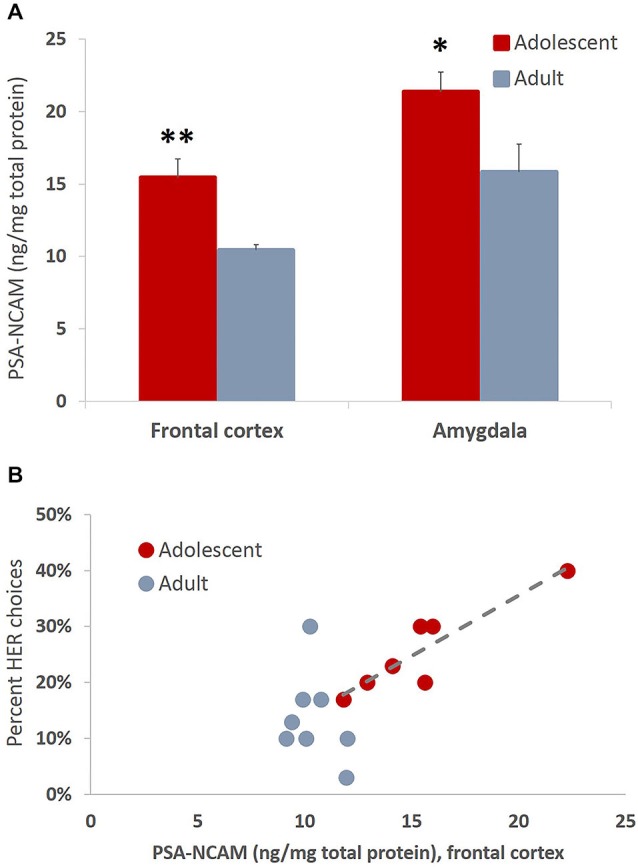
**Developmentally-specific patterns of PSA-NCAM expression are associated with greater effort expenditure toward larger rewards. (A)** The bar graphs represent the concentration of PSA-NCAM (ng/mg of total protein) + SEM. The levels of PSA-NCAM were increased in frontal cortex and amygdala in late adolescent compared to adult animals that had been trained on an effortful reward valuation task. **(B)** PSA-NCAM levels in PFC were strongly and positively associated with HER choices when considering the entire cohort of animals and in adolescents alone, but not in adult animals considered independently. The regression line is given for the adolescent group only *p < 0.05, **p < 0.01.

The present study revealed increased levels of PSA-NCAM in frontal cortex (*t*_(13)_ = 3.993, *p* = 0.002) and amygdala (*t*_(14)_ = 2.35, *p* = 0.034) of animals tested in adolescence as compared to animals tested in adulthood (Figure 8A). Interestingly the levels of PSA-NCAM in frontal cortex, not amygdala, were strongly and positively correlated with HER reward choices when considering the entire cohort of animals (*r*_(16)_ = 0.737, *p* < 0.01) and in adolescents alone (*r*_(7)_ = 0.909, *p* < 0.01), but not in adult animals considered independently (Figure 8B).

## Discussion

Preclinical research is aimed at understanding maladaptive behaviors induced by the detrimental effects of developmental experience, specifically with drugs of abuse and stressors. Suboptimal strategies may have a distinct meaning for adolescent and adult animals, depending on the temporal proximity of the reproductive period (Gluckman et al., 2007). For example, increased energy expenditure toward palatable foods may be suboptimal in adult animals that need to invest more time and effort in searching for potential mates or providing care and shelter for offspring. Conversely, foraging for nutritional rewards is critical for adolescent animals to ensure immediate survival, promote growth and increase reproductive fitness later in life. The present findings inform our understanding of behavioral phenotypes at different developmental stages. Specifically, we show that adolescent animals are indistinguishable from adults in appetitive associative learning, but exhibit distinct pattern of responses following a receipt of lesser-than expected outcome. Increased sensitivity to differences in outcome values was further supported by an enhanced motivation to invest effort to obtain larger rewards over less valuable, freely available options. Additionally, we report distinct expression patterns of frontocortical and amygdalar PSA-NCAM and striatal dopamine receptors depending on developmental period. Importantly, only frontocortical synaptic remodeling was associated with outcome valuation.

### Reward Learning is Similar in Adolescent and Adult Animals

Associative learning is highly important for many characteristic animal behaviors in the wild, including exploration of novelty, increased attention to change, and approach to potential rewards (Cloninger and Gilligan, 1987). An ability to learn the association between the appetitive outcomes and predictive stimuli provides an evolutionary advantage as it allows animals to maximize the rewards, of great importance to mammalian species (Bitterman, 1975). The results of the present investigation suggest that by the time rodents transition from complete reliance on their adult conspecifics and begin exploring their surroundings independently (i.e., adolescent period; Galef, 1981), they already possess associate reward learning skills. We observed no age differences in stimulus-reward and instrumental learning: adolescent animals were as fast as adults to master the operant task and direct their responses toward relevant stimuli. Similarly, they efficiently learned the spatial distribution of reward densities in the maze task and established stable choice behavior at a rate comparable to adults.

Although we did not examine reward learning earlier in development, the present data suggest that appetitive learning is established before adolescence. It needs to be noted, however, that one previous report demonstrated impaired odor-discrimination learning in adolescent compared to both juvenile and adult animals (Garske et al., 2013). In that task, which may be more ethologically-relevant for rodents than our visual task, animals were first trained to dig in a cup filled with unscented playground sand to obtain a palatable food reward, after which they were presented with two odorized cups only one of which contained the reward. Adolescent animals were slower to acquire this odor-association task, an effect that disappeared with pre-training during the juvenile period. Taken together, these results suggest that adolescent animals are not different on measures of simple appetitive reward learning; they were still able to acquire sand-reward association. However, rats displayed a limited ability to fine-tune cue representations and demonstrate learning difficulties when cues had more than one attribute. Previous reports also indicate compromised ability to behaviorally adapt to a change in operant contingencies and extinguish previously reinforced responding in adolescent animals (Sturman et al., 2010; Andrzejewski et al., 2011). However, similar to the present results, younger animals in both of these studies efficiently learned simple stimulus- and action-reward associations.

### Adolescent and Adult Animals Display Distinct Patterns of Responses to Outcome Values, which Manifest in Increased Effort Toward More Profitable Options

The only developmental differences observed in the operant task were in reward-seeking behavior and the response strategy following a receipt of lesser-than expected outcome. Adolescent animals were faster to respond on the subsequent trial after receiving a small reward, which happened in the minority of the trials, suggesting increased sensitivity to a violation of reward expectancy. Indeed, younger animals may be evolutionarily primed to be more sensitive to changes in their environment, as it allows to increase overall immediate profitability of the situation and prepare themselves for the future (Spear, 2000; Gluckman et al., 2007). Whereas there was a strong tendency for invigoration of response following lesser-than-expected outcomes in adolescent animals (possibly in an attempt to increase overall number of rewards collected), adult animals were slowed by such outcomes.

In accordance with their increased sensitivity to differences in outcome value, adolescent animals also displayed increased motivation to work for rewards of greater magnitude. Their choice behavior was characterized by increased effort expenditure toward larger rewards, while adult animals showed a clear pattern of reward devaluation as a function of increased barrier heights. The observed differences in choice preferences may be due to potentiated reactions to novel palatable foods in younger animals. Adolescents have been previously shown to display conditioned place preference, a measure of reward, to novelty; an effect that is absent in adults (Douglas et al., 2003). Additionally, adolescent animals are more sensitive to natural rewards, consume more sucrose solution and exhibit greater positive taste responses than their adult counterparts (Wilmouth and Spear, 2009). Alternatively, adolescents may be more sensitive to changes in unpredictable conditions in their habitat, which modulates effort expenditure (McNamara et al., 2013). Specifically, in the present study, both adult and adolescent animals were raised in a benign, nutritionally optimal environment, with food and water provided *ad libitum*, and were socially housed; they did not need to actively forage for rewards. The mismatch that was introduced as a result of short-term food restriction, may have had a more profound impact on adolescents compared to adults. These findings suggest that experiences during adolescence may have more potential adaptive significance than those encountered later in adulthood.

### Neurodevelopmental Correlates of Reward-Sensitive Endophenotype

Dopaminergic neurotransmission within the striatum has long been recognized as critical for incentive motivation and optimal response allocation to rewards (Berridge and Robinson, 1998; Salamone and Correa, 2002; Ostlund et al., 2011; Salamone et al., 2012; Richard et al., 2013). D1R and D2R density in the striatum peaks at the onset of the adolescent period, followed by extensive pruning to adult levels (Gelbard et al., 1989; Teicher et al., 1995; Tarazi and Baldessarini, 2000). The results of the present investigation revealed unaltered D2R expression, but reduced D1R expression in the striatum of animals that had experiences with reward learning during adolescence compared to animals that went through the same learning in adulthood. Decreased expression of D1R may result in diminished neuronal excitability in the striatonigral pathway upon dopamine release (Aosaki et al., 1998), and may ultimately lead to reduced learning from positive outcomes (Cox et al., 2015). It needs to be noted that because the brains were collected following training and establishment of stable performance, we are unable to distinguish age-specific from experience-dependent receptor expression profiles. However, previous reports indicate that D1R expression reaches mature levels by early adulthood (Teicher et al., 1995; Tarazi and Baldessarini, 2000). Therefore, reward experiences during adolescence may exaggerate normal pruning patterns and result in lower D1R levels as compared to the same experiences encountered in adulthood. Increased D1R expression early in adolescence (Gelbard et al., 1989; Tarazi and Baldessarini, 2000) may aid in establishing a pattern of behavior characterized by greater effort expenditure toward larger rewards, whereas decreased levels of D1R expression at the onset of adulthood would render animals less vulnerable to the effects of experiences with potent reinforcers.

Information transfer between amygdala and PFC has been shown to be critical for optimal reward-driven effort expenditure in maze tasks (Floresco and Ghods-Sharifi, 2007), with basolateral amygdala (BLA) signaling differences in reward magnitude (Salinas et al., 1993; Pratt and Mizumori, 1998). Cortical projections from BLA undergo remarkable development during adolescence (Casey et al., 2000; Cunningham et al., 2002, 2008; Brenhouse and Andersen, 2011). PSA-NCAM may play an important role in such structural and functional changes given its importance in activity-dependent synaptic remodeling and developmental events (Muller et al., 1996, 2010; Dey et al., 1999; Durbec and Cremer, 2001; Kiss and Muller, 2001; Welzl and Stork, 2003) resulting in prominent patterns of expression in regions undergoing active functional restructuring (Nacher et al., 2002a,b; Seki, 2002; Varea et al., 2005). Tsoory et al. (2008) reported significant decreases in PSA-NCAM expression with developmental progression from adolescence into adulthood in amygdala and hippocampus of naïve animals. The results of the present investigation revealed increased levels of PSA-NCAM expression in PFC and amygdala in late adolescent compared to adult animals that had been trained on an effortful reward valuation task. Intriguingly, PSA-NCAM levels in PFC were strongly and positively associated with HER choices in adolescents, but not in adult animals. To our knowledge, this is the first report showing a link between outcome valuation and developmentally-specific differences in PSA-NCAM expression. PSA-NCAM in the adult brain is restricted to interneurons, at least in PFC and BLA, and may aid in the incorporation of interneurons into circuitry to modulate local inhibition (Gascon et al., 2007; Gómez-Climent et al., 2011; Nacher et al., 2013). Increased levels of PSA-NCAM expression in adolescent animals in the present study may index increased structural plasticity within these brain regions and represent a neural correlate of a reward-sensitive endophenotype. However, additional investigations utilizing direct manipulations targeted to adolescent BLA and subregions within PFC are needed to establish a causal role for PSA-NCAM in adolescent-specific behavioral traits.

## Author Contributions

AS and AI designed research, analyzed data and wrote the paper; AS performed research.

## Conflict of Interest Statement

The authors declare that the research was conducted in the absence of any commercial or financial relationships that could be construed as a potential conflict of interest.
